# Development of a combined model incorporating clinical characteristics and magnetic resonance imaging features to enhance the predictive value of a prognostic model for locally advanced cervical cancer

**DOI:** 10.3389/fonc.2023.1284493

**Published:** 2023-11-22

**Authors:** Canyang Lin, Fengling Yang, Baoling Guo, Nan Xiao, Dongxia Liao, Pengfei Liu, Yunshan Jiang, Jiancheng Li, Xiaolei Ni

**Affiliations:** ^1^ Department of Radiation Oncology, The First Hospital of Longyan Affiliated to Fujian Medical University, Longyan, Fujian, China; ^2^ Department of Radiation Oncology, Fujian Medical University Cancer Hospital, Fujian Cancer Hospital, Fuzhou, Fujian, China

**Keywords:** cervical cancer, clinical characteristics, magnetic resonance imaging, prognosis, radiotherapy

## Abstract

**Objective:**

This study aimed to develop non-invasive predictive tools based on clinical characteristics and magnetic resonance imaging (MRI) features to predict survival in patients with locally advanced cervical cancer (LACC), thereby facilitating clinical decision-making.

**Methods:**

We conducted a retrospective analysis of clinical and MRI data from LACC patients who underwent radical radiotherapy at our center between September 2012 and May 2020. Prognostic predictors were identified using single-factor and multifactor Cox analyses. Clinical and MRI models were established based on relevant features, and combined models were created by incorporating MRI factors into the clinical model. The predictive performance of the models was evaluated using the area under the curve (AUC), consistency index (C-index), and decision curve analysis (DCA).

**Results:**

The study included 175 LACC patients. Multivariate Cox analysis revealed that patients with FIGO IIA-IIB stage, ECOG score 0-1, CYFRA 21-1<7.7 ng/ml, ADC ≥ 0.79 mm^2/s, and Kep ≥ 4.23 minutes had a more favorable survival prognosis. The clinical models, incorporating ECOG, FIGO staging, and CYFRA21-1, outperformed individual prognostic factors in predicting 5-year overall survival (AUC: 0.803) and 5-year progression-free survival (AUC: 0.807). The addition of MRI factors to the clinical model (AUC: 0.803 for 5-year overall survival) increased the AUC of the combined model to 0.858 (P=0.011). Similarly, the combined model demonstrated a superior predictive ability for 5-year progression-free survival, with an AUC of 0.849, compared to the clinical model (AUC: 0.807) and the MRI model (AUC: 0.673). Furthermore, the C-index of the clinical models for overall survival and progression-free survival were 0.763 and 0.800, respectively. Upon incorporating MRI factors, the C-index of the combined model increased to 0.826 for overall survival and 0.843 for progression-free survival. The DCA further supported the superior prognostic performance of the combined model.

**Conclusion:**

Our findings indicate that ECOG, FIGO staging, and CYFRA21-1 in clinical characteristics, as well as ADC and Kep values in MRI features, are independent prognostic factors for LACC patients undergoing radical radiotherapy. The combined models provide enhanced predictive ability in assessing the risk of patient mortality and disease progression.

## 1 Introduction

Cervical cancer is the fourth most common malignant tumor among women (1). In recent years, the incidence rate of cervical cancer has declined, but it still remains high. Due to the lack of effective screening in some areas, the mortality rate of young women with cervical cancer has increased (2). Cervical cancer staging is primarily based on the International Federation of Gynecology and Obstetrics (FIGO) staging or the International Union for Cancer Control (UICC) TNM staging (3, 4). Research suggests that different clinical stages are associated with varying prognostic outcomes, with 5-year survival rates of approximately 92%, 65%, and 17% for early-stage, locally advanced, and metastatic cervical cancer, respectively (5, 6). Despite receiving platinum-based concurrent chemoradiotherapy (CCRT) followed by intracavitary brachytherapy (7), nearly 40% of patients with locally advanced cervical cancer (LACC) still experience poor outcomes such as recurrence and metastasis (8, 9). Currently, there is a lack of ideal treatment methods for recurrence or metastasis (10). Additionally, radiotherapy or surgery often leads to side effects and sequelae that significantly impact the patients’ quality of life. These unsatisfactory results highlight the need for more refined diagnostic and treatment management approaches for cervical cancer.

General clinical characteristics, such as FIGO stage, lymph node metastasis, histopathology, tumor size, and age, are common prognostic factors for survival in LACC patients (11, 12). However, there are variations in clinical outcomes among patients with the same characteristics after treatment. This suggests the importance of identifying additional independent prognostic factors based on clinical features to improve the ability to predict patient recurrence and metastasis. Magnetic resonance imaging (MRI) is an important diagnostic tool for evaluating cervical cancer, offering the advantage of assessing overall tumor imaging characteristics (13). Traditional MRI techniques such as T1-weighted imaging (T1WI) and T2-weighted imaging (T2WI) provide detailed anatomical images (14, 15). With advancements in technology, emerging MRI techniques like diffusion-weighted MRI (DWI-MRI) and dynamic contrast-enhanced MRI (DCE-MRI) can provide physiological, metabolic, and functional information (16). Previous studies have demonstrated the potential value of MRI in predicting treatment efficacy and survival (17). Therefore, it is crucial to integrate clinical features with MRI imaging features to construct more accurate predictive models for guiding the treatment of LACC and improving prognosis.

The objective of this study is to analyze the clinical characteristics and MRI features of LACC patients receiving radical radiotherapy and construct an effective prognostic model for predicting long-term survival and disease progression.

## 2 Materials and methods

### 2.1 Patients

Between January 2012 and December 2020, a total of 175 patients diagnosed with histopathologically confirmed cervical cancer (stage IIA-IVA CC at diagnosis) were included in this retrospective study. The patients were restaged in accordance with the 8th edition of the FIGO system. To be eligible for inclusion, patients had to meet the following criteria: 1) histologically diagnosed with cervical squamous cell carcinoma or adenocarcinoma, and 2) undergone imaging examinations, such as pelvic MRI, blood routine examination, biochemical examination, and tumor marker examination, two weeks before treatment initiation. Patients were excluded if they: 1) had received any antitumor treatment prior to their initial evaluation, 2) did not complete the planned radical radiotherapy, or 3) had obvious MRI artifacts or were unable to undergo MRI examination. The retrospective study was approved by the Institutional Review Board of our hospital, and all patients provided informed consent to participate in the study.

### 2.2 Treatment strategy

All patients underwent a combination of External Beam Radiation Therapy (EBRT) and high-dose brachytherapy. The clinical target volume (CTV) for EBRT included the cervical mass, the entire cervix, uterus, part of the vagina, parametrium, and draining lymph nodes (internal iliac, external iliac, common iliac, and presacral). The prescribed dose to the CTV was 4860-5040 cGy in 27-28 fractions, and lymph nodes involved were considered for Simultaneous Integrated Boost (SIB) to a dose of 5670-6160 cGy in 27-28 fractions. After 20 fractions of EBRT, all patients received brachytherapy, with a dose of 2600-2800 cGy in 4 fractions (once weekly) delivered to the point A of the pelvic dose reference point. Concurrent chemotherapy regimens for these patients included weekly cisplatin (CDDP 40 mg/m2) for 6 cycles or cisplatin and taxane (CDDP 75 mg/m2 + paclitaxel 175 mg/m2) administered every 3 weeks for 2-3 cycles. Out of the 175 patients, 110 (62.9%) received cisplatin-based concurrent chemoradiotherapy (CCRT), while the remaining 65 patients (30.8%) received neoadjuvant chemotherapy.

### 2.3 Collection of basic patient information and clinical characteristics

Basic information and clinical characteristics of cervical cancer patients treated at our hospital were obtained from our hospital’s information management system and laboratory management system. Clinical features included age, family history of cancer, ECOG score, FIGO stage, general tumor classification, maximum tumor diameter, pathological type, pathological differentiation, carbohydrate antigen 125 (CA125), carcinoembryonic antigen (CEA), cytokeratin fragment antigen 21-1 (CYFRA21-1), hemoglobin, leukocyte count, serum albumin, CCRT, and others. All the mentioned data represent the baseline characteristics of patients before treatment.

### 2.4 MRI features acquisition

MRI scans were conducted within 2 weeks before the initiation of treatment. GE Signa HDI Echospeed 1.5T or Philips Achieva 3.0T superconducting MR scanners with an 8-channel bulk phase array coil were used, along with GE AW4.6 or Philips ISP V7 workstations. The conventional MRI parameters primarily included the apparent diffusion coefficient (ADC), volume transfer constant (Ktrans), rate constant (Kep), extracellular volume fraction (Ve), and plasma volume fraction (Vp). Tumor volume delineation in T2-enhanced images was performed by tracing the tumor area on the workstation for each slice using a trackball.

### 2.5 Statistical analysis

In this study, statistical analysis was conducted using SPSS (version 26.0) and R software (version 4.4.1). Univariate and multivariate Cox analyses were performed to identify effective prognostic variables for the model. The “Forest Map” software package was used to generate a forest plot. AUC curves were plotted using Medcalc software, and differences in AUC prediction performance were compared using the DeLong method. Model comparison was performed using the consistency index (C-index) and decision curve analysis (DCA) methods. A bilateral p-value of less than 0.05 was considered statistically significant.

## 3 Results

### 3.1 Screening of effective variables for clinical and MRI features

**Table 1** displays the baseline clinical and MRI features of the 175 LACC patients enrolled in this study. The median follow-up time was 59.5 months (range: 13.17-106.70 months). Among the 175 LACC patients, 35 (20.0%) succumbed to the disease, 14 (8.0%) experienced local recurrence, and 16 (9.1%) developed distant metastasis. Univariate analysis followed by multivariate COX analysis incorporating clinically significant variables revealed that ECOG score, FIGO staging, and CYFRA 21-1 were independent prognostic factors for both overall survival (OS) and progression-free survival (PFS) in LACC patients (as shown in **Table 2**). Moreover, MRI features such as ADC and Kep were also identified as independent prognostic factors for OS and PFS (as shown in **Table 3**).

**Table 1:** Basic information of 175 patients with cervical cancer.

Characteristics	No	percentage	Characteristics	No	percentage
**Age**			**CA125**		
≥60 years	76	43.40%	≥14.6U/ml	104	59.40%
<60 years	99	56.60%	<14.6U /ml	62	35.40%
**ECOG rating**			unknown	9	5.20%
0-1 points	103	58.90%	**Hemoglobin**		
2-3 points	72	41.10%	≥90g/L	155	88.60%
**BMI**			<90g/L	20	11.40%
≥24	55	31.40%	**Serum albumin**		
18.5-24	109	62.30%	≥37g/L	147	84.00%
<18.5	11	6.30%	<37g/L	28	16.00%
Pathological type					
SCC	165	94.20%	**CCRT**		
Adenocarcinoma	9	5.10%	Yes	110	62.90%
Adenosquamous carcinoma	1	0.70%	No	65	37.10%
**Tumor type**			**ADC**		
Rape blossom type	114	65.10%	≥0.79 mm^^2^/s	139	79.40%
Nodule type	52	29.70%	<0.79mm^^2^/s	36	20.60%
Endogenous type	9	5.20%	**Kep**		
**FIGO staging**			≥4.23min	59	33.70%
IIA-IIB	85	48.60%	<4.23min	105	60.00%
IIIA-IVA	90	51.40%	unknown	11	6.30%
**Tumor size**			**Ktrans**		
≥5.35cm	58	33.10%	≥1.23min	131	74.80%
<5.35cm	117	66.90%	<1.23min	33	18.90%
			unknown	11	6.30%
**Tumor volume**			**Ve**		
≥25cm^3^	83	47.40%	≥0.68	61	34.90%
<25cm^3^	92	52.60%	<0.68	103	58.80%
			unknown	11	6.30%
**CYFRA21-1**			**Vp**		
≥7.7ng/ml	27	15.40%	≥0.25	125	71.40%
<7.7ng/ml	138	78.90%	<0.25	39	22.30%
unknown	10	5.70%	unknown	11	6.30%

ECOG, Eastern Cancer Collaborative Group; FIGO, International Federation of Gynecology and Obstetrics; SCC, Squamous cell carcinoma; CYFRA21-1, Soluble Fragment of Cytokeratin 19; CA125, Carbohydrate Antigen 125; IMRT, Intensity Modulated Radiotherapy; CCRT, concurrent chemoradiotherapy; ADC, Apparent dispersion coefficient; Kep, Rate constant; Ktrans, Capacity transport constant; Ve, Percentage ratio of extracellular space volume; Vp, Plasma volume fraction.

**Table 2:** Univariate and multivariate COX analysis of clinical characteristics in cervical cancer patients.

Clinical characteristics	Univariate analysis (OS)		Multivariate analysis (OS)		Univariate analysis (PFS)		Multivariate analysis (PFS)	
	HR (95%CI)	*P*	HR (95%CI)	*P*	HR (95%CI)	*P*	HR (95%CI)	*P*
**ECOG**		0.001				<0.001		0.004
0-1 points	0.26		0.41	0.038	0.23		0.27	
2-3 points	(0.12-0.55)		(0.17-0.99)		(0.11-0.46)		(0.11-0.66)	
**FIGO stage**		0.001		0.038		0.002		0.037
IIIA-IVA	3.55		2.4		3.03		2.4	
IIA-IIB	(1.66-7.58)		(1.05-5.51)		(1.50-17.0)		(1.05-5.24)	
**Tumor size**		0.015		0.95		0.011		0.297
≥5.35cm	2.28		0.97		2.29		0.66	
<5.35cm	(1.19-4.48)		(0.39-2.45)		(1.21-4.33)		(0.30-1.45)	
**Tumor volume**		0.025		0.94		0.008		0.628
≥25cm3	2.2		0.96		2.69		1.12	
<25cm3	(1.11-4.38)		(0.32-2.85)		(1.30-5.57)		(0.56-2.64)	
**CYFRA21-1**		<0.001		0.008		<0.001		0.003
≥7.7ng/ml	5.32		3.24		6.66		3.28	
<7.7ng/ml	(2.64-10.9)		(1.35-7.78)		(3.37-13.2)		(1.49-7.26)	
**CA125**		0.004		0.094		0.011		0.302
≥14.6U/ml	0.33		0.54		3.12		1.51	
<14.6U/ml	(0.16-0.70)		(0.26-1.11)		(1.30-7.49)		(0.69-3.30)	
**CCRT**		0.039		0.83		0.027		0.964
Yes	0.48		0.91		0.48		0.92	
No	(0.24-0.96)		(0.40-2.09)		(0.25-0.92)		(0.45-2.14)	
**Serum albumin**								
≥37g/L					0.45	0.031	0.56	0.302
<37g/L					(0.21-0.93)		(0.27-1.19)	

**Table 3:** Univariate and multivariate COX analysis of MRI features in cervical cancer patients.

MRI features	Univariate analysis (OS)		Multivariate analysis (OS)		Univariate analysis (PFS)		Multivariate analysis (PFS)	
	HR (95%CI)	*P*	HR (95%CI)	*P*	HR (95%CI)	*P*	HR (95%CI)	*P*
**ADC** ≥0.79 mm^2/s <0.79mm^2/s	0.384	0.006	0.414	0.02	0.44	0.017	0.44	0.019
	(0.19-0.77)		(0.20-0.87)		(0.23-0.86)		(0.23-0.87)	
**Kep** ≥4.23min <4.23min	0.374	0.029	0.35	0.021	0.41	0.033	0.36	0.016
	(0.15-0.9)		(0.14-0.85)		(0.18-0.93)		(0.16-0.83)	
**Ktrans** ≥1.23min <1.23min	2.28	0.121	2.1	0.276	2.71	0.059	2.37	0.162
	(0.80-6.45)		(0.55-7.93)		(0.96-7.64)		(0.71-7.95)	
**Ve** ≥0.68 <0.68	0.639	0.194	0.61	0.245	0.73	0.334	0.93	0.826
	(0.325-1.257)		(0.26-1.41)		(0.39-1.38)		(0.49-1.78)	
**Vp** ≥0.25 <0.25	2.25	0.093	1.25	0.712	2.62	0.044	1.52	0.455
	(0.87-5.80)		(0.38-4.08)		(1.01-6.70)		(0.51-4.56)	

### 3.2 Multifactor forest plot of clinical and combined MRI features

**Figure 1:**
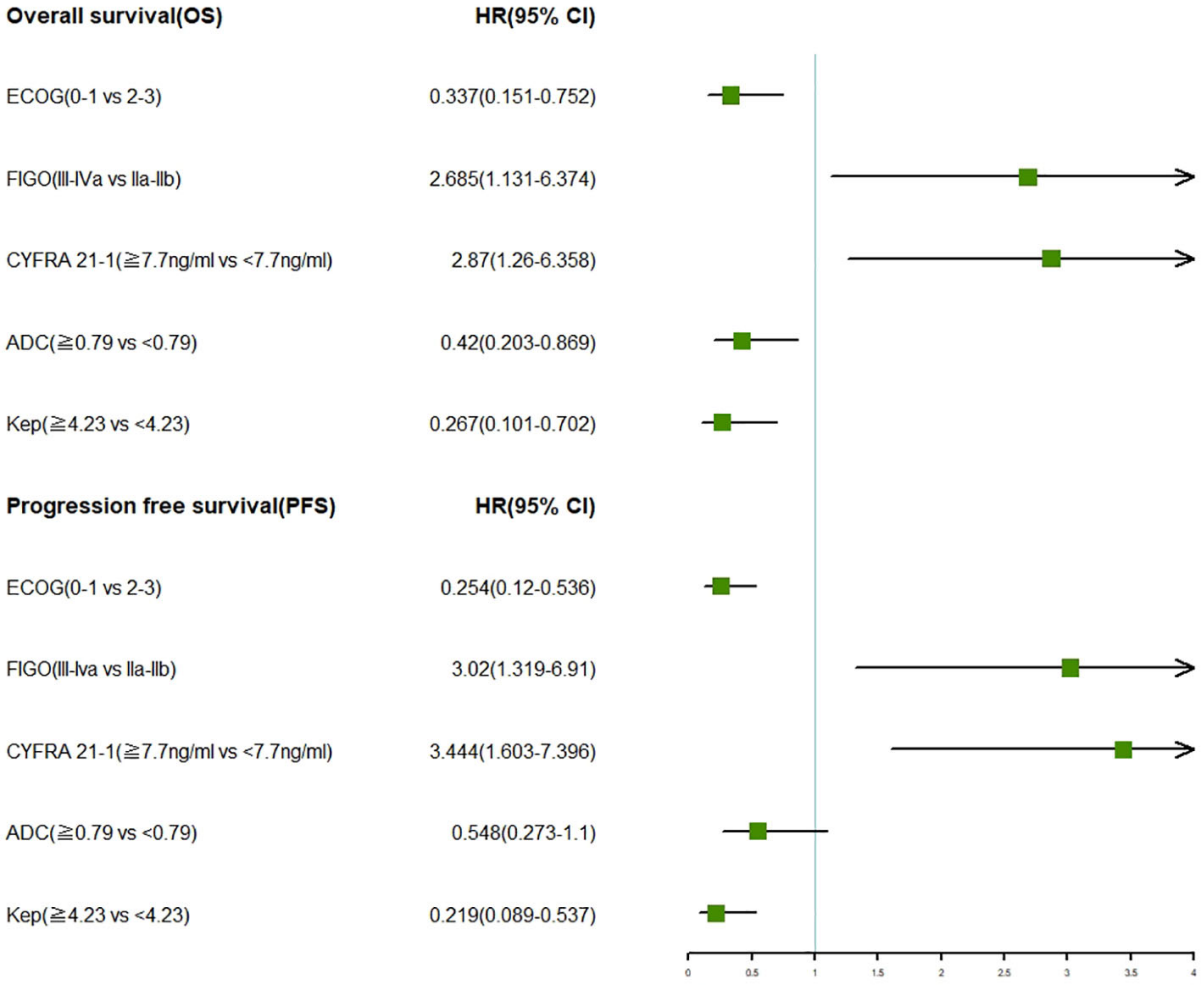
Forest plots showcasing multivariate analysis of clinical characteristics and MRI features in relation to OS and PFS in LACC patients.

The multifactor forest plot resulting from the multivariate analysis included three clinical features (ECOG score, FIGO staging, CYFRA 21-1) and two MRI features (ADC, Kep). Regardless of OS or PFS, patients with LACC who had FIGO stage IIA-IIB, ECOG score 0-1, CYFRA 21-1 < 7.7 ng/ml, ADC ≥ 0.79 mm^2/s, and Kep ≥ 4.23 min exhibited more favorable survival outcomes, as indicated by the multivariate COX analysis (**Figure 1**).

### 3.3 Construction of a prognostic prediction model based on clinical characteristics and MRI features

#### 3.3.1 Clinical model

**Figure 2:**
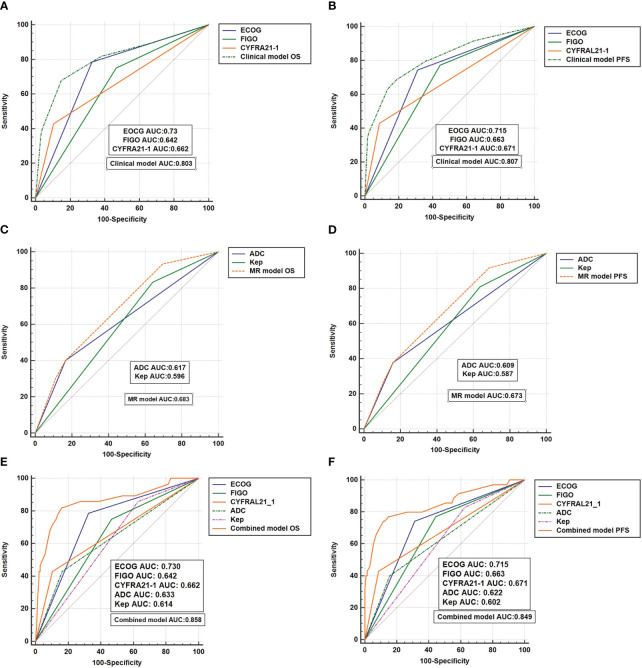
The ROC curves **(A, B)** reflect the efficacy of clinical models in predicting OS and PFS; **(C, D)** reflects the effectiveness of the MR model in predicting OS and PFS; **(E, F)** reflects the effectiveness of the combinated model in predicting OS and PFS.

The clinical model, incorporating ECOG score, FIGO staging, and CYFRA 21-1 as independent prognostic factors, was developed to predict 5-year OS and PFS. The clinical model exhibited an AUC value of 0.803 for predicting 5-year OS, which outperformed the individual prognostic factors, namely CYFRA 21-1 (AUC: 0.662), FIGO staging (AUC: 0.642), and ECOG score (AUC: 0.73) (P < 0.05). Similarly, the clinical model achieved an AUC value of 0.807 for predicting 5-year PFS, surpassing the individual prognostic factors (CYFRA 21-1: AUC 0.671, FIGO staging: AUC 0.663, ECOG score: AUC 0.715) (P < 0.05). The corresponding curves are illustrated in **Figures 2A, B**.

#### 3.3.2 MRI model

The MRI model, based on independent prognostic factors ADC and Kep, was developed to predict survival outcomes. The MRI model demonstrated an AUC of 0.683 for 5-year OS, outperforming the individual prognostic factor ADC (AUC: 0.617) (P = 0.0065) and exhibiting a trend compared to Kep (AUC: 0.596) (P = 0.09). Additionally, the MRI model predicted a 5-year PFS AUC of 0.673, which surpassed the individual prognostic factor ADC (ADC: 0.609) and showed a trend compared to Kep (AUC: 0.587) (P = 0.0078 for ADC and P = 0.068 for Kep). Please refer to **Figures 2C, D** for the corresponding curves.

#### 3.3.3 Combined model

By integrating the clinical model and the MRI model, a combined prediction prognosis model (the combination model) was developed. The combined model yielded a 5-year OS AUC of 0.858 for LACC patients, which was higher than the AUC of the clinical model (0.803) (P = 0.0099) and the MRI model (0.683) (P = 0.0109). Similarly, the combined model predicted a 5-year PFS AUC of 0.849, surpassing the clinical model (AUC: 0.807) (P = 0.0032) and the MRI model (AUC: 0.673) (P = 0.0056). **Figures 2E, F** display the corresponding curves. .

### 3.4 Model prediction performance comparison

**Figure 3:**
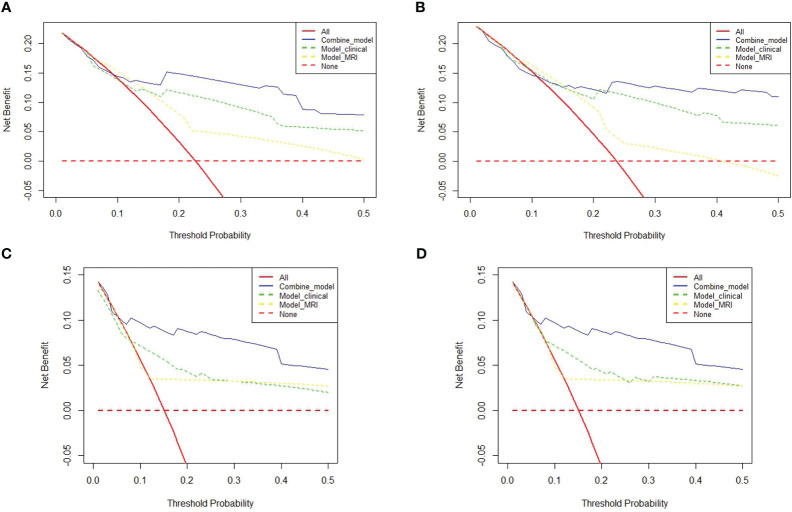
Decision Curve Analysis (DCA) plots: **(A, B)** contrast the predictive efficacy for OS and PFS among different models for 175 LACC patients. **(C, D)** delineate the comparative efficacy of different models in predicting OS and PFS in a subset of 110 LACC patients undergoing concurrent radiotherapy and chemotherapy (CCRT).

The prognostic prediction models were further evaluated using the C-index. The combined model achieved a C-index of 0.826 (0.761-0.890) for predicting OS survival in LACC patients, which was superior to the clinical model (0.763, 0.685-0.841) and the MRI model (0.665, 0.585-0.745). For PFS, the combined model demonstrated a C-index of 0.843 (0.78-0.90), outperforming the clinical model (0.8, 0.735-0.865) and the MRI model (0.646, 0.568-0.724). Please refer to **Table 4** for detailed results. Additionally, the decision curve analysis (DCA) results, as depicted in **Figures 3A, B**, illustrate the superior predictive performance of the combined model compared to the clinical and MRI models for both OS and PFS.

**Table 4:** Comparison of C-index among different prognostic prediction models.

Prognostic prediction model	OS		PFS	
	C-Index1* (95% CI)	C-Index2** (95% CI)	C-Index1* (95% CI)	C-Index2** (95% CI)
Clinical Model	0.763 (0.685-0.841)	0.886 (0.723-1.00)	0.8 (0.735-0.865)	0.906 (0.809-1.00)
MR Model	0.665 (0.585-0.745)	0.75 (0.481-1.00)	0.646 (0.568-0.724)	0.781 (0.659-0.902)
Combined Model	0.826 (0.761-0.890)	0.977 (0.839-1.00)	0.843 (0.78-0.90)	0.969 (0.91-1.00)

C-index1* is the C-index of the prognosis model of 175 patients with cervical cancer; C-index2** is the C-index of the prognosis model of 110 patients with cervical cancer receiving concurrent chemoradiotherapy.

### 3.5 Subgroup analysis

**Figure 4:**
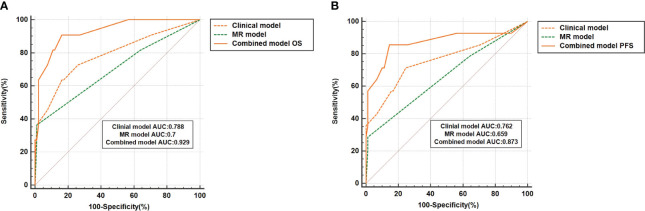
The ROC curve represents the performance of different prognostic models for 110 LACC patients who received concurrent radiotherapy and chemotherapy (CCRT). **(A, B)** represent the results of the model's prediction of OS and PFS, respectively.

This study delved deeper into a subgroup analysis of 110 LACC patients who underwent concurrent chemoradiation (CCRT). **Figures 4A, B** reveal that the 5-year OS AUC for the combined model stood at 0.929, surpassing both the clinical model (AUC: 0.788) and the MRI model (AUC: 0.7). Similarly, the combined model’s 5-year PFS AUC was 0.873, outdoing the clinical (AUC: 0.762) and MRI (AUC: 0.659) models. Among the 110 LACC patients treated with CCRT, the C-index for OS was 0.886 (0.723-1.00), which was better than both the clinical model’s 0.886 (0.723-1.00) and the MRI model’s 0.75 (0.481-1.00). Regarding PFS, the combined model’s C-index was 0.969 (0.91-1.00), outperforming the clinical model (0.906, 0.809-1.00) and the MRI model (0.781, 0.659-0.902). Comprehensive results can be found in **Table 4**. Additionally, the DCA results (**Figures 4C, D**) suggest that the combined model surpassed the clinical and MRI models in predicting OS and PFS for LACC patients undergoing CCRT.

## 4 Discussion

Despite concurrent chemoradiotherapy (CCRT) treatment, approximately 40% of patients with locally advanced cervical cancer (LACC) still face the risk of recurrence or metastasis (18). This underscores the need for more accurate prognostic models in clinical practice. Our research demonstrates that ECOG score, FIGO staging, and CYFRA21-1 are independent prognostic factors for cervical cancer patients. The clinical model constructed using these factors exhibits superior predictive performance compared to any single factor, facilitating the identification of LACC patients with a poor prognosis. Furthermore, we have incorporated magnetic resonance imaging (MRI) factors such as ADC and Kep into the clinical model to develop a combined model. The results indicate that the combined model surpasses both the clinical and MRI models in predicting 5-year overall survival (OS) or progression-free survival (PFS) and C-index in LACC patients. Additionally, Decision Curve Analysis (DCA) demonstrates that the inclusion of MRI factors in clinical models significantly enhances predictive performance.

Accurate staging plays a crucial role in determining treatment strategies, estimating prognosis, and guiding follow-up for malignant tumors. The revised FIGO cervical cancer staging standard in 2018 introduced imaging and pathology options (19). It divided stage IB into three groups based on tumor diameter and added a new stage IIIC to reflect the survival heterogeneity associated with lymph node involvement (4). Tang et al. (20) conducted a retrospective analysis of 3,238 cervical cancer patients, revealing FIGO staging as an independent prognostic factor for 5-year survival, with a C-index value of 0.721 indicating good predictive ability. Consistently, our study demonstrates that FIGO staging is an independent prognostic factor for 5-year OS and PFS in LACC. Patients in stages IIIA-IVA exhibit a 2.4 times higher risk of death and disease progression compared to those in stages IIA-IIB. Moreover, unlike previous studies, our research incorporates factors such as ECOG score, tumor markers, and MRI imaging.

The ECOG score serves as a scale to assess whether cancer patients’ physical condition can tolerate anti-tumor therapy (21). Research suggests that ECOG scores are associated with chemotherapy response, tolerance, survival rates, and quality of life (22). However, limited studies have explored the correlation between ECOG scores and long-term survival in cervical cancer. Our study reveals the ECOG score as an independent prognostic factor for long-term survival in LACC patients undergoing radical radiotherapy. Patients with an ECOG score of 0-1 demonstrate better 5-year OS and PFS compared to those with a score of 2-3 (P<0.05). Prior studies have indicated a correlation between pre-treatment CYFRA21-1 levels and tumor size, staging, and worse pathological classification (23, 24). In our study, we determined the optimal cutoff value for CYFRA21-1 to be 7.7 umol/l, with patients having ≥7.7 umol/l exhibiting worse 5-year OS and PFS (P<0.001). The clinical model constructed based on the three clinical factors—ECOG score, FIGO staging, and CYFRA21-1—outperforms any single prognostic factor, achieving an AUC of 0.803 for 5-year OS and 0.799 for 5-year PFS.

Magnetic Resonance Dynamic Contrast-Enhanced Imaging (DCE) employs swift, recurring enhancements to evaluate tumor vascular permeability and perfusion, offering a prognostic assessment for cancer patients (25). Mayr et al. (26) identified tumor heterogeneous regions with diminished DCE values, correlating with a risk of treatment failure, using DCE functional MRI. They also quantified the functional risk volume (FRV). The findings point out that FRV is an innovative functional imaging heterogeneity metric, superior to the anatomical tumor volume (ATV). It holds the potential for clinical transition into personalized early outcome forecasts before treatment initiation or as early as 2-5 weeks post-treatment. This might clarify why the ATV didn’t hold significance in our multivariate analysis, leading to its exclusion from the model analysis. Beyond FRV, contemporary research highlights DWI-based ADC, K(el), and Ktrans as stand-alone prognostic indicators for LACC patients (14) (27). In line with these insights, our investigation underscores that both DWI ADC and DCE Kep values are pivotal prognostic elements for the extended survival of LACC patients. Nevertheless, our research boasts a considerably larger cohort and an extended overall monitoring duration compared to preceding investigations. Furthermore, unlike certain prior studies, we’ve integrated clinical characteristics, especially those linked with radiation therapy, for collective scrutiny and juxtaposition.

Yu W et al. (28) gathered data on 13,802 LACC patients from the SEER database and developed a prediction model using a machine learning (ML) algorithm to estimate the 5-year survival of LACC patients. Their findings indicated that the XGBoost model displayed the most superior predictive capability, achieving an AUC of 0.8365 — a performance that surpassed both the LR and SVM models. In our research, when we incorporated MRI indicators into the clinical model, we noted an enhancement in the AUC for the 5-year OS predictive capacity of the combined model to 0.858 (P=0.011), marginally outperforming the XGBoost model. Similarly, the combined model registered a 5-year PFS AUC value of 0.849, overtaking the clinical model’s 0.807 and the MRI model’s 0.673. As further validated by the DCA curve analysis, the combined model consistently exhibited the most commendable predictive accuracy. Additionally, a noteworthy limitation of the XGBoost model is its omission of a clear delineation regarding the treatment approach for LACC. Variances in treatment strategies can considerably influence cervical cancer outcomes. Consequently, we executed a subgroup analysis focusing on 110 LACC patients who underwent CCRT. This deep dive revealed AUC values for the 5-year OS and 5-year PFS from the combined model as 0.886 and 0.969, respectively. This methodology stands out as it proficiently discerns patients facing elevated risks of recurrence and mortality.

There are several limitations to our study. Firstly, it is a retrospective study, and rigorous prospective cohort studies are needed for further validation. Secondly, external validation from other institutions is necessary to confirm our findings. Lastly, our study includes a limited number of MRI parameter items. Therefore, our future research will focus on incorporating additional multi-parameter imaging features, such as imaging omics features, to establish a more effective prognostic prediction model for LACC.

## 5 Conclusion

Clinical factors, including ECOG score, FIGO staging, and CYFRA21-1, are independent prognostic factors for LACC patients, with the clinical model exhibiting superior predictive power compared to individual factors. Furthermore, the addition of MRI factors to the clinical model to construct a combined model yields the best predictive performance, providing valuable guidance for clinical decision-making.

## Data availability statement

The raw data supporting the conclusions of this article will be made available by the authors, without undue reservation.

## Ethics statement

The studies involving humans were approved by Ethics Committee of Longyan First Hospital. The studies were conducted in accordance with the local legislation and institutional requirements. The participants provided their written informed consent to participate in this study. Written informed consent was obtained from the individual(s) for the publication of any potentially identifiable images or data included in this article.

## Author contributions

CL: Writing – original draft, Conceptualization, Formal analysis, Investigation, Software, Supervision, Writing – review & editing. FY: Data curation, Writing – original draft. BG: Writing – original draft, Formal analysis, Methodology. NX: Methodology, Writing – original draft. DL: Investigation, Writing – original draft. FY: Writing – original draft. PL: Writing – original draft. YJ: Writing – original draft. JL: Conceptualization, Writing – review & editing. XN: Writing – original draft, Writing – review & editing.
